# Nurses’ experiences of encounters in home care: a phenomenological hermeneutic study

**DOI:** 10.1080/17482631.2021.1983950

**Published:** 2021-10-11

**Authors:** Anna Larsson Gerdin, Ove Hellzén, Malin Rising-Holmström

**Affiliations:** Department of Nursing Sciences, Mid Sweden University, Sundsvall, Sweden

**Keywords:** Encounters, home care nursing, lived experience, narrations, nurse-patient relationship, vulnerability

## Abstract

**Purpose:**

nurses working in home care often encounter patients with multiple diagnoses in unpredictable environments. This may cause ethical and emotional challenges and influence nurses’ daily work. The aim of this study was to illuminate the meaning of nurses’ lived experiences of encountering patients in home care.

**Methods:**

narrative interviews were conducted with 11 nurses. These interviews were audiotaped and transcribed verbatim and analysed using a phenomenological hermeneutic approach.

**Findings:**

the findings are presented under three main themes: (1) “Being receptive to the other” (with subthemes “Caring about the encounter,” and “Establishing trusting relationships”). (2) “Handling the unpredictable” (with subthemes “Being alone in the encounter” and “Being experienced and competent”). (3) “Managing frustration” (with subthemes “Feeling insufficient” and “Feeling restricted”. Having overall nursing responsibility challenged the nurses’ self-confidence in providing care trustfully.

**Conclusions:**

encountering patients in home care means relating to the other unconditionally, which aim to highlight patients’ needs. Being a nurse in home care is both emotionally demanding and rewarding. Having the courage to face their own and the patients’ vulnerabilities will entail the promotion of natural receptivity and responsiveness to patients’ needs.

## Introduction

Working in home care, a nurse will encounter patients throughout their lives. Although there is no age limit for receiving home care in Sweden, older persons are in the majority, and many of them are frail and with multiple diagnoses (Johansen & Fagerström, 2010). This is often combined with a high symptom burden of pain, depression, and fatigue, among many others (Eckerblad et al., 2015). This leads to increasingly advanced health- and medical care being carried out in the home (Nilsson et al., 2009).

The focus of care for elderly has been transferred from hospitals to a model of home care (Carlson et al., 2014; Turjamaa et al., 2014), with municipalities having overall responsibility to organize and provide nursing measures that follow the Health and Medical Services Act (SFS 2017:30) and the Social Services Act (SFS 2001:453). In Sweden, home care is defined as “health care when it is given in the patient’s home or equivalent, and where the responsibility for medical measures is consistent over time” (The National Board of Health and Welfare, 2020). Home care in the Nordic countries is unique as it offers services to all citizens in need, regardless of family situation, network, or income (Turjamaa et al., 2014), and is being granted based on a lack of ability to get to a healthcare centre (Bökberg & Drevenhorn, 2017). The overall objectives of home care are to improve quality of life and maintain independence by assessments of people’s individual needs and measures taken (Thomé et al., 2003).

In Sweden, registered nurses are given responsibility for caring for people in their homes. Within home care, nurses have the highest level of education and are legally responsible for the care provided (SFS 2017:30). Nurses perform a variety of tasks including assessment, planning, and evaluating the outcome of activities such as bandaging, blood and urine tests, giving medication by injection, and terminal care (Genet et al., 2012; Karlsson et al., 2013). Fewer hospital beds and more outpatient care means that home care even encompasses follow-up care and rehabilitation (Garåsen & Hendriksen, 2009).

Depending on their needs, patients may be cared for over several years in their homes, meaning that nurses often develop long-term relationships with them (McGarry, 2003). In this encounter, interpersonal interaction takes place. This is based on the attitudes people have towards each other and is shown in the way someone acts or behaves towards others in the form of gestures, words, and measures. Encounters are affected by previous experiences and existing expectations before the encounter (SOU 1995:159), and are influenced by nurses’ ability to convey a sense of accessibility and commitment (Öhman & Söderberg, 2004). A close, trusting relationship in home care is important, as it may increase feelings of well-being and promote patients’ health in their daily lives (Corbett & Williams, 2014). This is achieved by making it easier for people to adapt to new circumstances and increasing their collaboration with the nurse, and may also lead to an increased sense of security (Leslie & Lonneman, 2016).

Providing nursing care in someone’s home means an intrusion into the patient’s privacy is inevitable (Magnusson et al., 2002). Moreover, encountering patients in their homes is not the same as encountering them within the safe territory of a hospital. When entering the patient’s home, there are expectations from both sides which affect social interaction (Holmberg et al., 2012). These expectations may be complicated as they may be experienced differently (Lindahl et al., 2011). For example, a nurse entering someone’s home may feel they are doing so as a guest (Öresland et al., 2008), while the patient may experience the nurse as a professional who is only there to perform a task in the patient’s best interest. It is important to balance these expectations because good collaboration with the patient is required. After all, the nurse’s workplace is in the patient’s home, which forms the basis of security concerning personal privacy (Holmberg et al., 2012).

Caring for the patients in their homes puts nurses in a vulnerable position as they experience a variety of consuming emotions and stresses in their work (Stenbock-Hult & Sarvimäki, 2011). It is of the utmost importance to understand the emotional and ethical dimensions of encountering patients in home care, and pay more attention to how it impacts the way nurses practice their profession. Studies focusing on the meaning of nurses’ lived experiences of these kinds of encounters, especially in the Swedish context is sparse. Thus this study has aimed to illuminate the meaning of nurses’ lived experiences of encountering patients in home care.

## Method

### Design

This study is qualitative in design and adopts a phenomenological hermeneutic approach. The approach was inspired by Ricoeur’s (1976) interpretive theory, which was further developed as a research method by Lindseth and Norberg (2004). The purpose of using this method was to reveal the meaning of individuals’ everyday experiences by interpreting texts transcribed from lived narratives. The approach combined the phenomenological goals of describing and explaining lived experiences with the hermeneutical goals of understanding and interpreting the same. The method provides an opportunity to increase understanding of nurses’ encounters with patients in home care.

#### Procedure and setting

Since knowledge from the Swedish home care context in which the phenomenon is studied is sparse, a homogeneous selection strategy has been used and data has been collected from a specific defined group, which will provide a deeper understanding of the essential meaning of nurse-patient encounter in a homecare context. A purposive sample was employed to recruit registered nurses (RN) with the inclusion criterion of having experienced encountering patients in home care. Permission to recruit participants from the home care team was obtained by the senior manager. Oral information on the study was given, and a letter with an invitation to participate was handed out in person and emailed to all RNs working in home care (n 18). This letter contained written information about the study, a consent form, and a stamped return envelope addressed to the first author. Eleven RNs gave their consent to participate in this study and all were contacted by telephone to make interview appointments. The sample size was guided by the concept of information power developed by Malterud et al. (2016), which means that the more information the sample holds, the fewer participants are required. Following Malterud et al.’s (2016) criteria, 11 participants were an acceptable sample size to reach information power. The study setting was a municipality in northern Sweden with approximately 25,000 inhabitants. This consisted of rural areas, and two urban areas close to the only city in the east of the county. The municipal was served by one home care team with a central base treating about 500 patients per year.

#### Data collection

Data was collected through individual narrative interviews with open-ended questions (*cf*. Mishler, 1986), to capture lived experiences of encounters in home care (Lindseth & Norberg, 2004). The interviews were conducted from March to May 2020 by the first author, and in accordance with the recommendations on social distancing due to the COVID-19 the interviews were conducted through phone. The interviews ranged from 70–90 minutes (md 80 min) and were audiotaped and transcribed verbatim. The first author had some experience working in home care, but was not familiar with the participants. The interviews began with informal small-talk so as to create a respectful, friendly atmosphere. The participants were then asked to answer three main questions:

“Please tell me about an everyday encounter with a patient in home care”.

“Please tell me about an encounter with a patient that evoked negative feelings”.

“Please tell me about an encounter with a patient that evoked positive feelings”.

For clarification and to encourage deeper exploration of lived experience, supplementary questions were used, such as “please tell me more”, “what did you do then?” and “how did you feel?”

#### Phenomenological-hermeneutic approach

The phenomenological hermeneutic method (Lindseth & Norberg, 2004) means moving between the whole text and parts of that text using three interrelated phases: naive understanding, structural analysis, and comprehensive understanding.

In the first phase, the analytical process started with narrative interviews which were read several times by the first author to grasp the initial meaning of the text. This led to a naïve understanding of the meaning of the nurses’ lived experiences of encountering patients in home care. In the next phase, a structural analysis took place, to identify parts and patterns and seek clarification of the text by outlining units of meaning and condensing them into everyday language. All authors further abstracted and organized the condensed meaning units into subthemes, with similar meanings identfied and sorted into themes. Each theme and subtheme were named and, in the findings, illustrated with quotations from the interviews. All phases in the structural analysis included a continuous reflection on the initial naïve reading of the text and the aim of the study. The analytical process was repeated until the naïve understanding was validated by the structural analysis. As a final phase, a comprehensive understanding was devised as a critical, in-depth interpretation, following the text from what it said to what it talked about. The naïve understanding, the findings of the structural analysis, the authors’ pre-understanding, relevant theories, and previous research were all brought together in the comprehensive understanding, that aimed at a deeper interpretation of the text and deeper understanding of the studied phenomenon (Lindseth & Norberg, 2004). The naïve reading, structured analysis and comprehensive understanding are presented under findings.

#### Ethics

Ethical considerations consistently followed the research ethics rules under the Declaration of Helsinki (World Medical Association , 2018), and ethical approval to perform the research was obtained from the Swedish Ethical Review Authority, (Dnr. 2019–06333). Even though the nurses provided written consent, participation was voluntary, and they could cease participation at any time without giving a reason. The participants were guaranteed confidentiality and only the researchers connected with the project had access to the data, as per the guidelines of The Swedish Research Council (2017).

## Findings

The study participants were all RNs working in home care, seven of whom had a master’s degree in nursing. Their work experience as RN ranged from one to 40 years (md 17.9 y), and work experience of working in home care ranged from five months to 10 years (md 4.4 y). In this study, all RNs will be referred to as nurses, regardless of their educational level.

### Naïve understanding

During their working day, the home care nurses encountered patients with varying needs. These encounters made different impressions on the nurses, and even though such encounters were usually perceived as positive, some were more challenging. For example, when the nurse took actions that were not always appreciated by the patient, but necessary for good care. These encounters resulted in feelings of sadness, irritation, and frustration. The challenges in these encounters became particularly prominent when nurses felt limited due to lack of knowledge and information, patient compliance, and work environment. To balance the demands emerging in the encounter, nurses assumed different roles and strategies. This facilitated flexibility and an opportunity to establish a good relationship. Encounters that went smoothly were perceived as positive and made nurses feel affirmed. However, nurses also experienced encounters characterized by shortcomings and feelings of powerlessness, discomfort, and resignation. Experience, competence, and receptivity facilitated the unpredictability and challenges that sometimes emerged in home care encounters and contributed to good collaborations between sole nurses and their patients.

### Structured analysis

Three themes and six subthemes emerged from the structural analysis of the text, see Table I. These reflected the essential meanings of the phenomenon “nurses’ encounters with patients in home care”. The presentation of the findings is written in the past tense and illuminates what Ricoeur (1976) calls “utterance meaning”. The utterance was separated from the author in that it was fixed in text, and thus the meaning of the text could be described.

**Table I.** Overview of themes and subthemes

Being receptive to the other	Caring about the encounter Establishing trusting relationships
Handling the unpredictable	Being alone in the encounter Being experienced and competent
Managing frustration	Feeling insufficient Feeling restricted

### Theme 1: Being receptive to the other

The theme “Being receptive to the other” illustrates the meaning of being open minded, taking your time and acknowledging the patient. Encountering patients in their homes meant becoming a part of the patients’ whole being and connecting to the unique reality that belongs to the person. This theme includes the subthemes “Caring about the encounter” and “Establishing trusting relationships”.

### Caring about the encounter

Encountering the patient at home meant that a nurse had to adapt to a patient’s situation. Caring about the encounter meant creating the conditions to become receptive to the patient’s conveyed needs. For example, by inviting the patient to the conversation. Humbly taking the time to listen and become aware of the patient’s situation meant that what the patient conveyed became clearer.

“You must perceive the situation and assess what I can talk to this person about. I have to ask myself: How can I make the person feel confident in me and feel safe with me? How should I do that? What kind of person do I have in front of me; like, what does the person want? What does the person need? But it is difficult … ”

(Participant 6)

Sometimes the nurses felt they were not able to care about the encounter, like when there was a lack of time which entailed an increased risk that the encounter became forced. This was hard to handle and aroused inner stress in nurses. The nurses emphasized the importance of remaining calm and objective in these encounters. Their feelings and thoughts were curbed so as to perceive the patient’s wishes and needs.

“With empathy and understanding, you’ll go far. Just listening again and letting them tell the same story every time you’re there.”

(Participant 9)

### Establishing trusting relationships

The nurses emphasized the importance of having a trusting relationship so as to support the patient. Having a relationship and experiencing the patient’s trust meant that a nurse could feel safe, secure, and receptive to their patient’s conveyed needs in the encounter, this gave the nurse energy. To establish relationships, nurses assumed different roles. For example, they used the same wording as the patients when describing things and used humour and jokes when the patient did the same. In this way, the nurse tried to encounter the patient respectfully on the patient’s conditions.

* “Some patients I approach calmly. Others… I remember a patient who was so dear to me. There was fun and jokes from the very first minute. I always start the dialog with something fun, and then I had his full trust.”*

(Participant 1)

In encounters where a nurse was emotionally touched, their receptivity to their patient’s perceived situation improved. The nurse’s desire to care increased, as it confirmed their feelings of commitment. In encounters where the patient was perceived as unpredictable, unpleasant, and distancing, the nurse’s ability to establish relationships became more difficult, and their desire to care was reduced. Sometimes this led to the nurse trying to avoid visiting the patient by exchanging visits with a colleague. If a nurse, despite everything, did manage to reach the patient, it strengthened the nurse.

“*When you’ve found your way in there, when you’ve passed this little, almost rebellious ‘No, no, no, no,’ and found a way in, then you can find a topic of conversation or something. This means they’ve accepted me, accepted my presence in their lives. Then it actually feels really good!”*

(Participant 9)

### Theme 2: Handling the unpredictable

The theme “Handling the unpredictable” illustrates the meaning of encountering the unknown as a sole nurse in the patient’s home. During their day of work, nurses usually encounter their days through what they plan and control. However, handling the unpredictable means acknowledging the patients’ differences and being prepared for the unforeseen in every encounter. This theme includes the subthemes “Being alone in the encounter” and “Being experienced and competent”.

### Being alone in the encounter

Being alone in the encounter with a patient could lead to feelings of insecurity in interpreting and understanding what the patient conveyed. Nurses tried to perceive their patients’ situation by reading between the lines. However, there was heightened uncertainty during the initial encounter. As there was no previous relationship or overall picture of the patient’s situation, the first encounter was sometimes perceived as uncertain, raising concerns about not correctly assessing the patient’s care needs.

“*It’s different if you have a relationship than when you only visit the person now and then. It means you get to know each other! But if it’s the first time you’re seeing this seriously ill person, then the encounter can be a bit difficult. I have to be aware of how I feel and respond to them in a difficult situation.”*

(Participant 5)

Encountering the patient alone fed into a perceived nervousness before each challenge. As a result, the nurses might perceive their own behaviour and actions as unnatural, uncomfortable, and rigid. This might lead to a reduced ability to inspire security and confidence, with the nurse sometimes feeling a need to prove their knowledge to maintain trust. Being alone when encountering the patient meant being in a vulnerable position, which culminated when risking one’s safety.

“S*ometimes you can feel, “no, there probably should be two of us in here!” Because you know there’s a history of violence. And in that situation, I can’t really feel comfortable. It’s actually quite unpleasant!”*

(Participant 4)

### Being experienced and competent

Working in home care required broad skills as nurses often ended up in unpredictable situations. Through the experience of managing the patient’s needs, nurses’ competence, and awareness of their way of working grew.

“It requires experience and security to work in home care because you’re so alone. When you go to an individual’s home, that you’ve sort of seen different things before. But I also meet those I have great difficulty in assessing. But when you’ve been working for a while, your sense of security does grow.”

(Participant 11)

This increased professional experience meant that nurses became more accustomed to and confident in seeing the patient’s whole situation. This was even more important when quick assessments were needed. A nurse’s previous experience contributed to an increased ability to handle the encounter regardless of any challenges, which was valuable in the next encounter.

“*I feel safe and secure in myself, and have no fear or discomfort visiting any patient. I also feel that I can go to anything myself because I know that I have so much experience and feel safe.”*

(Participant 3)

Based on previous encounters, nurses developed strategies for dealing with similar situations in the future, which reduced stress and insecurity in the encounter. Nurses found new ways to express themselves and act, which led to them feeling more competent in handling unpredictable encounters.

“Now I know how to manage. If I see his moped at home, he is home. If the moped is not at home, then I know that he has left and I have to return later. We have built a relationship, so I know what time I can return to him and knock on the door. In this case, he is so anxious that I cannot unlock the door and enter. I have to knock and wait until he opens. I cannot do it any other way. But now I feel safer.”

(Participant 11)

### Theme 3: Managing frustration

The theme “Managing frustration” illustrates the meaning of managing your emotions and highlighting the needs of the patients. It includes feelings of shortcomings concerning what nurses want and what they can do for the patient. This theme includes the subthemes “Feeling insufficient” and “Feeling restricted”.

### Feeling insufficient

Feeling insufficient meant being aware of one’s own limitations in one’s role as a nurse. If the nurses felt limited, there was a risk of perceived inadequacy in their work. For example, when an assessment did not yield results which could lead to self-blame: *“Could I have done it any other way, or did I do enough?”* This could mean a bad conscience as the nurse could not respond to the conveyed need as they would have liked.

“It was so hard! And I scrutinized myself every day when I was … If there was anything else I could say or do. I even offered to go with him to the hospital. But I didn’t succeed. No, I don’t know. He was in my mind for a very long time.”

(Participant 1)

Working in home care involved responsibility for striving to improve the patient’s situation. When there were difficulties fulfiling patient expectations, nurses became aware of the setbacks that may emerge when caring for patients at home. Feelings of insufficiency could evoke feelings of frustration, failure, and powerlessness. *“You feel like ‘oh I can’t even manage this!’ That I’ll have low self-esteem the next time I go there.”* Managing these feelings was about deciding to try and resolve the situation or resigning to accepting it the way it was.

“Well, of course, that will affect you because I really couldn’t do any more for her other than be there for a while. And I think that feels so sad. But I need to let go of it.”

(Participant 10)

### Feeling restricted

Feeling restricted meant balancing between following laws and rules with being responsive and respecting the patient’s right to self-determination. Maintaining this balance meant being challenged in one’s commitment to the encounter. According to the nurse, this led to a perceived ambivalence about how the patient should be cared for. This ambivalence was expressed, for example, through anger and frustration regarding patients with dementia:

“Yes, I get frustrated! All those people with dementia who can’t cope. They have to stay at home alone in their apartments. And they’re hallucinating, feeling scared and worried, and in pain, and exposed.”

(Participant 2)

Sometimes nurses performed measures that the patient wanted but that the nurse did not assess to be in the patient’s best interests. For example, the nurses might perceive that the patients made poor judgements in refusing treatment. Such occasions could be hard to handle, and the nurses had to rely on careful persuasion. In these situations, nurses had to struggle to handle their own reactions to respecting the patient’s choice, and this affected the nurses’ conscience.

“I’m thinking of a patient who also lived quite far away. Who didn’t agree to what I thought he needed. I tried to do the best I could at home. But I realized that this wouldn’t hold, as the patient required hospital care. But I got no further. That made me feel very frustrated and sad.”

(Participant 1)

## Comprehensive understanding

In the reflection on the naive reading and themes within the findings, it appeared that encountering the patient in home care meant being emotionally touched and facing vulnerability in oneself as well as in the patient. Understanding and balancing the empathetic dimension in care causes confusion and anxiety, which challenges the role of nurse and might lead to the state of being generous or being distant towards the patient. Encountering patients in home care involved an effort in getting to know patients and their experiences by being sensitive and receptive to what patients conveyed. By being attentive to and responding to patients’ needs, the nurses’ vulnerability emerged that they solely must handle. Our interpretation suggests that managing vulnerability will enhance the nurse’s ability to encounter the patient in home care in a perceptive way.

The aim of this study was to illuminate the meaning of nurses’ lived experiences of encountering patients in home care. Three themes emerged which shed light on nurses’ lived experiences: “Being receptive to the other”, “Handling the unpredictable” and “Managing frustration”.

The theme “Being receptive to the other” meant caring about the encounter and striving to establish trusting relationships. Caring about the encounter meant that nurses became aware of and accepted the vulnerability of the patient’s situation. It was characterized by the nurse being perceptive to the need of the other, which meant seeing the “person” and not just the “patient”. The home care nurses encountered patients daily who were experiencing suffering related to vulnerabilities caused by their illness or disability. In occasions, nurses did not have the time to perceive more than the measures they were there to perform. This can be understood as a problem since when health care is required, a patient’s vulnerability becomes more than ordinarily according to Sellman (2005), and having a protective function in regard to patient’s vulnerability is definitely a legitimate and fundamental part of the role of nurses. The findings highlighted that in being attentive to what patients convey, nurses must deal with both their own and their patients’ vulnerability. Without being attentive, patients risked being overlooked, as also expressed by Eilertsen and Kiik (2016). Furthermore, nurses’ inability to care about the encounter and truly see the patient might cause suffering rather than alleviation (Svanström et al., 2013), which according to our findings led to emotional challenges as the nurses could not always care about the encounter as much as they wanted.

Patient narrations constitute was the starting point for the relationship. Moreover, the nurses’ ability to listen is important in terms of mutual trust. Findings from this research capture how encountering patients in home care can mean relating to the other person unconditionally and spontaneously, in response to their situation. Previous studies illuminate the fact that to perceive a patient’s need for care in an everyday context and create a mutual understanding of the lived situation, nurses must pay attention to the expressed narrations (Ekman et al., 2011). By taking patients’ problems seriously and making a visible commitment to help, nurses create trust (Tarlier, 2004; Van Hecke et al., 2011). This is developed through communication, a sense of community, and reciprocity (Leslie & Lonneman, 2016). As the patients’ vulnerability is a main issue in nursing, and the nurses’ vulnerability lies in their engagement in caring for the patients (Angel & Vatne, 2017), a relationship built on trust will enable nurses to be where the patients are (Truglio-Londrigan, 2013).

The findings highlight how encounters with reluctant patients in home care might have a negative effect on nurses and that they may become insecure and feel inadequate and powerless. The findings could be understood as a risk for not providing adequate support based on need as it will get more difficult if the patient turns against the nurse, also described in earlier research by Angel and Vatne (2017).This was more common in the encounters where patients are not compliant, passive, cooperative and participatory (Molina-Mula & Gallo-Estrada, 2020). By being emotionally touched and having the courage to face their own vulnerability and that of their patients, nurses’ desire to care grew and attempts to avoid visiting by exchanging visits with colleagues were prevented.

Another way of understanding the phenomenon of encountering patients in home care may be “Handling the unpredictable”. This meant handling encounters alone in the patients’ home and being experienced and competent. Encountering patients in unpredictable situations was sometimes stressful and emotionally demanding, and the nurses were exposed as they had to improvise and face issues alone. Being in a vulnerable position, encountering patients alone, sometimes meant fear of not doing the right thing. The findings indicates that when nurses had no prior knowledge or relation to the patient, the encounters in home care were unpredictable, and it was difficult to determine and assess the best care for the patient in those encounters. The findings illuminates that misinterpreting patient needs features as a concern for the nurses; one which caused their consciences to be troubled. Öhman and Söderberg (2004) explain that these findings might emerge from assuming the heavy responsibility of having a central role as sole home care nurse. It also corresponds to Hendry and Walker (2004), who describe home care of patients as complex. Having the opportunity to plan and prioritize is significant as it will allow the potentially severe consequences of confusion, disorganization, and poor patient care to be counteracted. Sørensen and Hall (2011) claim that to handle the unpredictable, nurses must seek the bigger picture. To not overlook patient needs which require immediate measures, nurses must connect the threads into a web of care and accomplish their integrative role working in home care.

By acknowledging their vulnerability in unpredictable situations meant that nurses’ competence was sometimes questioned by patients and by the nurses themselves. Feelings of doubt emerged as to whether the nurses were good enough to assess measures which might improve their patients’ quality of life and support them in maintaining independence. The findings highlighted how the ability to recognize cues and interpret unpredictable situations was facilitated by previous knowledge, experience, education, and cognitive strategies. This was also described by Hendry and Walker (2004) and Kihlgren et al. (2006), which adds the nurse’s role perception as an important factor. It has been stated that the ability to provide competent care is not merely a question of age and postgraduate education (Karlstedt et al., 2015), but a combination of knowledge, performance, skills, and attitudes (Cowan et al., 2005). Evolving in the role of a nurse in home care takes time and is referred to by Sneltvedt et al. (2010) as a lonely process since nurses work alone in patients’ homes. Decisions have to be made in situations where there are only limited colleague support and guidance available. In the findings, these decisions are facilitated by feeling secure in encountering the unpredictable.

The theme “Managing frustration” refers to nurses facing moments of distress, not knowing how to balance patient rights with the nurses’ own professional commitment. These moments aroused feelings of insufficiency and demonstrated the importance of maintaining a balance between internal and external demands to not feel restricted. The nurses were assuming a responsibility that challenged the ethical dimension of caring. Feeling insufficient meant an inability to fulfil the notion of always being able to alleviate a patient’s suffering. This was reinforced where efforts were made, but resulted in no improvement. It becomes a source of suffering to which the nurse has no access and thus cannot alleviate.

The findings can be understood as a risk to having a troubled conscience. This includes, according to Ericson-Lidman et al. (2013), having to accept situations that felt wrong and having to act against patients’ will. Feelings of insufficiency might arise from not being able to reach patients or interpret their needs and wishes, and having to rely instead on guesswork (Sundström et al., 2018). Nurses in home care must assess measures that are more or less agreeable to patients and may be why they perceive a failure to meet their expectations of professional nursing (Samia et al., 2012). Conscience seems to be closely connected to the self, and thus going against one’s conscience is naturally associated with high levels of stress (Glasberg et al., 2008).

The findings in this study refer to encounters within home care as a balancing act, meaning that nurses must handle conflicting emotions to counteract a sense of failure leading to emotional distress. One example involved an ambivalence whereby the nurses felt they had not provided adequate care as they felt restricted by patients own will or regulations. This was related to feelings of not doing the right thing and could lead to the nurses not meeting their patients’ expectations, diminishing the nurses’ trust in their own abilities. As described by Breitholtz et al. (2013), it may be difficult for a nurse to choose between avoiding, confronting, and mediating when guided by laws, regulations, templates, and decisions about patients’ care needs since they seek to do what is right. Avoiding conflict means a risk of distancing themselves from the patient if they do not know how to act in a given situation. The findings illuminate how nurses used persuasion and compromise as strategies concerning patients they found challenging to encounter. Rasoal (2020) explain these strategies as finding a balance between different expectations and that careful persuasion is a positive strategy to deal with these expectations. On the other hand, Michaelsen (2012), states that these strategies might increase the risk of a nurse becoming emotionally stagnant, as they concentrate on the physical aspects of illness and miss the patient’s view of their situation in context of their lives.

Further understanding of the various dimensions of meaning in this phenomenon may be gained from Danish philosopher Knud Eijler Løgstrup’s (1994) description of ethical demand. Løgstrup’s thinking was primarily about the creative theological anchoring of charity, the silence of the ethical demand, and the ethics of moral responsibility. Being human means being exposed to each other and living with various challenges and demands in a life of interdependence, of which vulnerability is a fundamental condition. Our findings indicate that caring about the encounter means nurses adapting to their patients’ situation and perceiving what is unspoken. Caring for a patient who touches them emotionally makes nurses more open to patient vulnerability and it becomes easier to respond to patient needs.

In addition to being silent and unspoken, the ethical demand is also unilateral, spontaneous, and radical (Løgstrup, 1994). This means that nurses in home care will meet and respond to a demand as they perceive it, regardless of the patient’s wishes and expectations. According to the findings of this study, making unilateral decisions in unpredictable situations based on nurses’ pre-understanding and abilities increases the nurses’ vulnerability. There are challenges in how nurses (due to their vulnerability, their interpretation of the situation, and their engagement in the encounter) are able to be open and receptive to the other person, with nurses sometimes facing moments of distress not knowing how to meet demands due to them being unattainable. To avoid abusing power in the relationship, Løgstrup (1994) stated that nurses must do what is best for the other person. The ethical demand reaches a deeper level than “right and wrong.” Regardless of external demands, the nurses must perceive, interpret, and decide what is best for the patient, and they are responsible for making the outcome of the encounter as good as possible.

The findings of this study illuminate the fact that the framework of assuming different roles is a way of encountering patients individually. It enhances the ability to perceive the ethical demand unconsciously sent out by the other person, while the nurse’s ability to interpret and perceive a situation enables them to care without violating. Martinsen (2006) states that to avoid making decisions that negatively affect patient vulnerability, nurses create frameworks; a silent set of rules derived from previous encounters and experiences of clinical practice. As the ethical demand is spontaneous and sprung from the given conditions of human coexistence (Løgstrup, 1994), the frameworks will enable nurses in home care to act in unpredictable situations. Løgstrup (2018) states that the immediate and spontaneous demand presupposes control of the state of mind and expression. This means that nurses must hide their true feelings and moods behind a fictional persona.

The findings highlights how, by having the courage to face their own vulnerabilities and those of their patients, nurses will discover a genuine interest in the other. It expresses through attempts to understand their patients’ lived experience, which encourages the nurses to care. Løgstrup (1994) claimed that to not betray the other person, the unilateral ethical demand requires nurses to care for the life of the patient unselfishly. We are all in each other’s power because our vulnerability and solitude will facilitate a natural trust. A trust that we hope will be answered and fulfilled through measures that touch, and show care and affection.

In conclusion, encountering patients in home care is challenging as well as rewarding in many ways. Encounters in home care involve a great deal of responsibility, in that nurses act alone and cannot always meet unattainable demands. Behind every illness, there is a person who needs to be seen, meaning that the nurse must move the focus from efficiency and solutions and try get to know the persons in front of her, perceiving their needs and wishes. Lack of time and a pressured schedule could mean that the nurse tended to miss what the patients conveyed and therefore wasn´t able to live up to the ethical demand. By encountering a vulnerable patient group, the nurse’s vulnerability is waken. This vulnerability needs to be highlighted and reflected on, as it is valuable for not becoming cynical.

## Methodological considerations

By reflecting on the naive understanding, and the explanation of structural analysis, as expressed in relation to ones’ own pre-understanding and previous scientific literature, a new and deeper understanding of the phenomenon was obtained. The design of this study is suitable for revealing the meaning of individuals’ everyday experiences and a purposive sample is an adequate method of recruiting from the available population, as it meets the inclusion criteria. To the best of the authors’ knowledge, no previous studies have focused on this phenomenon in the context of home care. Therefore, a homogeneous sampling strategy was preferable, rather than a more varied and heterogeneous selection. This was because it provided a deeper understanding of the phenomenon being studied. According to Patton (2015), qualitative research strives to study phenomena in natural contexts, for which reason lived experience from nurses encountering patients in home care was requested. Gathering data from only one home care group may be a limitation. However, trustworthiness is strengthened by truthful narrations (Lindseth & Norberg, 2004). The fact that the interviewer was not known by the interviewees might enable them to narrate freely and truthfully, without fear of revealing weaknesses.

Narrated text has several meanings and may be understood and interpreted in different ways (Lindseth & Norberg, 2004; Ricoeur, 1976). The findings presented represent what was considered the most credible understanding of the meaning of lived experience, with the text re-contextualized. By analysing narrations using a phenomenological hermeneutic approach (going from part to whole in the hermeneutic spiral) will validate the process (Ricoeur, 1976). The first author conducted all the interviews, transcribed the text, and conducted the initial analysis. However, since it is possible to interpret text in different ways (Ricoeur, 1976), all three authors, each with different nursing experience, reflected on and continuously and critically worked on the assessments until a consensus was reached. The authors’ pre-understanding of the context was diverse and was reflected throughout the analytical process. This increased the ability to bracket and perceive implicit messages.

Valid quotations present and can lend increased credibility to the analysis by minimizing the risk of misunderstanding (Ricoeur, 1976). It should be born in mind that, due to transferability, home care in Sweden is municipal and its uniqueness may differ nationally. This might be perceived as a weakness of this study. The current findings should, therefore, be seen as lived experiences in the Swedish context. However, these findings may be relevant in other contexts, for enriching the understanding of the complex encounters between nurses and patients. Hopefully, the study’s findings will encourage more research into the emotional and ethical dimensions of encountering patients in home care.
